# On the Relationship Between Muscle Synergies and Redundant Degrees of Freedom in Musculoskeletal Systems

**DOI:** 10.3389/fncom.2019.00023

**Published:** 2019-04-16

**Authors:** Reza Sharif Razavian, Borna Ghannadi, John McPhee

**Affiliations:** Motion Research Group, Department of Systems Design Engineering, University of Waterloo, Waterloo, ON, Canada

**Keywords:** motor control, muscle synergy, task space, redundant motion, feedback control, fast control, uncontrolled manifold, orthogonal basis vectors

## Abstract

It has been suggested that the human nervous system controls motions in the task (or operational) space. However, little attention has been given to the separation of the control of the task-related and task-irrelevant degrees of freedom.

**Aim:** We investigate how muscle synergies may be used to separately control the task-related and redundant degrees of freedom in a computational model.

**Approach:** We generalize an existing motor control model, and assume that the task and redundant spaces have orthogonal basis vectors. This assumption originates from observations that the human nervous system tightly controls the task-related variables, and leaves the rest uncontrolled. In other words, controlling the variables in one space does not affect the other space; thus, the actuations must be orthogonal in the two spaces. We implemented this assumption in the model by selecting muscle synergies that produce force vectors with orthogonal directions in the task and redundant spaces.

**Findings:** Our experimental results show that the orthogonality assumption performs well in reconstructing the muscle activities from the measured kinematics/dynamics in the task and redundant spaces. Specifically, we found that approximately 70% of the variation in the measured muscle activity can be captured with the orthogonality assumption, while allowing efficient separation of the control in the two spaces.

**Implications:** The developed motor control model is a viable tool in real-time simulations of musculoskeletal systems, as well as model-based control of bio-mechatronic systems, where a computationally efficient representation of the human motion controller is needed.

## 1. Introduction

Two of the major complexities associated with the human motor control system are: (1) The number of degrees of freedom in the human body greatly exceeds the minimum number required to finish a task. (2) Each degree of freedom is affected by multiple muscles that need to cooperate in order to perform the movement. As a result, different movement patterns can accomplish a given task (the *degree of freedom problem*, Bernstein, 1967), and a specific movement can be generated by an infinite number of muscle activation combinations (the *muscle redundancy problem*). From a mechanical point of view, the first is a kinematic redundancy, while the second is a dynamic redundancy.

It has been observed that the movements exhibit stereotypical features in the task (or operational) space. Morasso (1981) has shown that in reaching tasks, the hand follows a straight line from the origin to the target, and the hand velocity profile is stereotypically bell-shaped. However, no such consistency could be observed in joint angle trajectories. Furthermore, the uncontrolled manifold theory (UCM, Scholz and Schöner, 1999) also theorizes that the nervous system actively controls the task-related degrees of freedom, and leaves the rest uncontrolled. These observations support the existence of a control mechanism in the task space. However, there are situations when not just the task-related variables, but all the degrees of freedom need to be actively controlled (e.g., reaching a target with a specific hand orientation). How do these situations fit in the “task space control” theme? In addition to that, it is not clear how the nervous system may control the muscles (that essentially rotate the joints) to selectively control some kinematic variables, and leave the rest uncontrolled. In this paper, we propose a computational framework that can achieve such a selective control scheme.

Numerous computational models for the control of musculoskeletal systems have been proposed. Among these, many direct optimization-based models exist (Todorov et al., 2005; Liu and Todorov, 2009; Mehrabi et al., 2015a,b, 2017) that inherently control all the degrees of freedom at all times, and as a result are computationally costly. Another challenge to these optimization methods is the choice of the objective function, which is the topic of *inverse* optimal control (searching for the correct objective function, Mombaur et al., 2010; Laschowski et al., 2018; Berret et al., 2019). The feedback control models developed by Park and Durand (2008), Blana et al. (2009), and Jagodnik and van den Bogert (2010) are joint space controllers that do not separate task-related and redundant kinematic variables. Lockhart and Ting (2007) and Sharif Razavian et al. (2015) have developed feedback controllers using modular activation of muscles, but for systems without kinematic redundancy. Stanev and Moustakas (2017) have employed a task-space formulation and an optimization routine to solve for the muscle activations. Fu et al. (2015) have developed a controller for a kinematically redundant system; however, only the control of the task-variables are reported. The only available computational framework that formulates the kinematic and dynamic redundancies in musculoskeletal systems is developed by Stanev and Moustakas (2019); however, no direct relationship between the muscle redundancy and kinematic redundancy is discussed.

Muscle synergy theory was originally proposed as a possible mechanism employed by the nervous system to reduce the number of control signals (Tresch et al., 1999; Tresch and Jarc, 2009). According to this theory, the nervous system builds the muscle activation commands, by combining a few sets of activation (called modules, muscle synergies, or motor primitives). Such a low-dimensionality in muscle activation signals has been observed in a variety of cases, e.g., healthy humans movements (Kutch et al., 2008; Meyer et al., 2016; Sharif Shourijeh et al., 2016; Smale et al., 2016), spinal cord injury (Zariffa et al., 2012), stroke (Cheung et al., 2009, 2012; Clark et al., 2010; Roh et al., 2013; Scano et al., 2017), and cerebral palsy patients (Steele et al., 2015; Tang et al., 2015), frogs (Cheung et al., 2005; Bizzi et al., 2008), and cats (Ting and McKay, 2007; Sohn and Ting, 2016). Muscle synergies are especially appealing from a computational point of view, as the dimension reduction in the control space contributes to the computational efficiency of the control algorithms for musculoskeletal systems.

The relationship between the muscle synergies and the task space is not fully studied. It has been shown that there is a correlation between the synergies and the endpoint force in Bizzi et al. (1991) and Ting and Macpherson (2005). Berger and D'Avella (2014) have used a mapping to estimate the end-point force from the measured muscle activities. Conversely, Lockhart and Ting (2007) have used the center of mass kinematics (task variable) to estimate muscle activations in a balancing task. Sharif Razavian et al. (2015) have proposed a mathematical relation between the dimensions of the task space and the number of muscle synergies required to control the task. There is one missing point in these articles: how do the synergies affect the redundant degrees of freedom, besides the task space?

In previous research (Sharif Razavian et al., 2015, 2019), we proposed a real-time motor control framework that takes advantage of the explicit relationship between muscle synergies and the task space forces, to control the movement in musculoskeletal systems. This framework could effectively control the movements in the task space, while leaving the redundant ones uncontrolled. The objective of the present work is to explore the potential of this framework for the selective control of some or all of the degrees of freedom in kinematically redundant musculoskeletal systems.

We start with a brief summary of the proposed motor control framework. Next, we show how the framework can be generalized to facilitate the control of redundant degrees of freedom. The experimental methods to evaluate the feasibility of the framework is presented next. In the end, the results and discussion are provided, followed by the concluding remarks.

## 2. Methods

According to the uncontrolled manifold theory (Scholz and Schöner, 1999), the nervous system tightly controls the task-related kinematic variables, and leaves the unrelated ones uncontrolled. There is an important complication inherent to this theory; the task variables (e.g., hand position in a reaching task) are in general complex functions of all the joint angles. How does the nervous system activate the muscles (which rotate the joints) to control some of the kinematic variables and leave the others uncontrolled?

**Figure 1:**
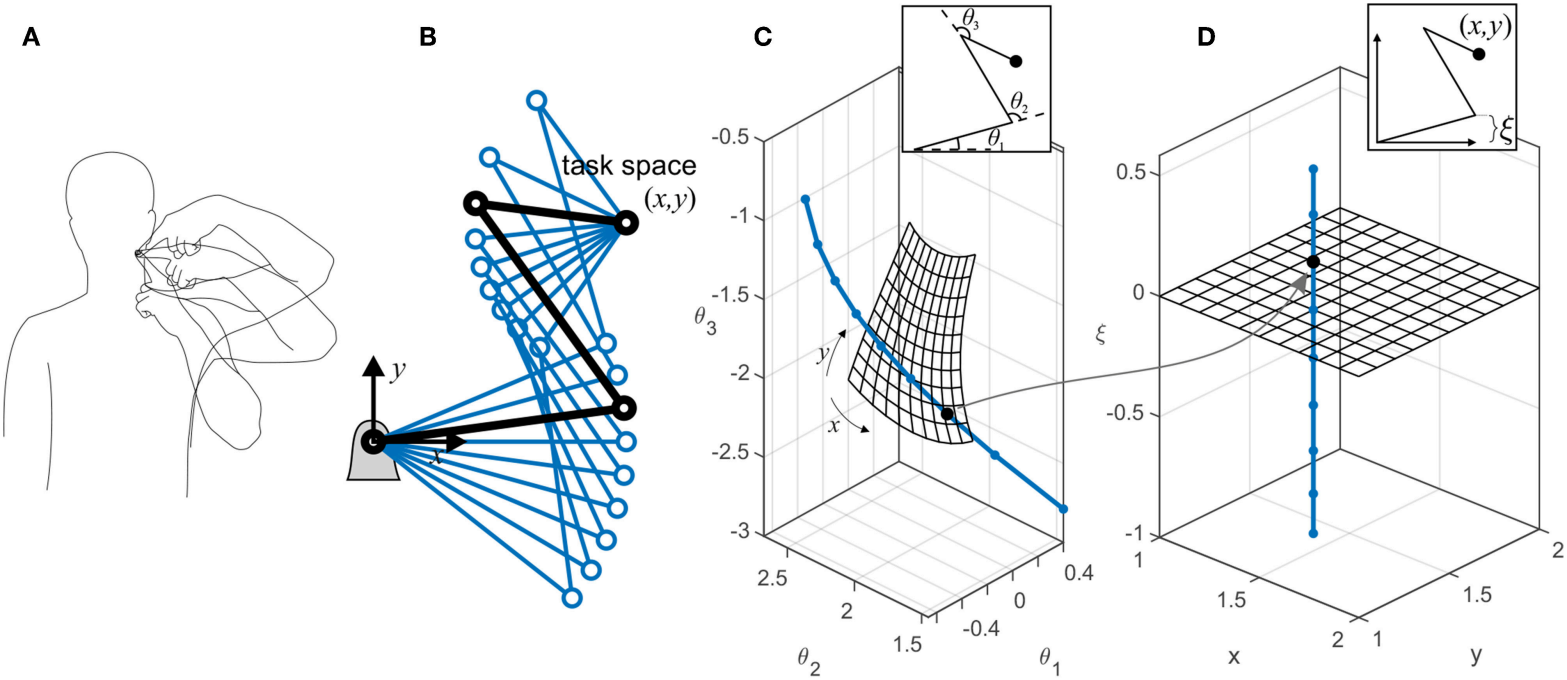
**(A)** There are multiple ways to perform a certain task. The variables that are related to the task (e.g., finger position in space) form the *task space*, while the ones that can change without affecting the task (e.g., elbow height) form the *redundant space*. **(B)** An example of a planar robotic system with kinematic redundancy. The robot has three degrees of freedom and a 2D task space (*x, y*). Because there is one extra (redundant) degree of freedom besides the task-related ones, the robot can reach the target in multiple configurations. **(C)** The representations of the robot in the joint space; each dot on the curved line represents a single configuration, all of which result in the same target position (*x, y*). The meshed surface represents the robot configuration at multiple target positions (with the constraint of θ_1_ = 0). **(D)** An equivalent representation of the robot in task/redundant space. The mesh surface represents the same target positions. In this example, the redundant variable ξ is chosen to be the y-component of the first link's end point [ξ = *l*_1_sin(θ_1_)]. The orthogonality of the task space (mesh) and the redundant space (dotted line) is evident. The dots show equivalent configurations of the robot in the two representations.

We use the term *task space* to describe the collection of the task-related variables that are actively controlled by the nervous system. Depending on the task requirement, these variables could be kinematic (e.g., finger position) or dynamic (e.g., pinch force). Without losing generalizability, we only discuss kinematic task variables here. For example during a reaching task, the task variables are the (*x, y, z*) position of the hand, and the task space is the 3D Cartesian space. Conversely, the collection of the kinematic variables whose variations do not affect the task form the *redundant space* (see Figure 1A). It is readily apparent that the redundant variables are functions of all the joint angles. However, it is possible to define generalized kinematic variables that are in a subspace orthogonal to the task space. Figure 1B shows an example of a 3-degree-of-freedom (3-DoF) system, which can be described by three coordinates, such as (θ_1_, θ_2_, θ_3_) or (*x, y*, ξ). The former is the joint angle representation (Figure 1C), while the latter is a task/redundant variable representation (Figure 1D). The variable ξ is a generalized coordinate that can be any linear or non-linear function of the joint angles, which along with the task variables (*x, y*) will uniquely define the system's configuration. Because ξ can vary without affecting the task variables, it lies in a subspace that is orthogonal to the task space.

We have introduced a computational motor control model based on a task space formulation in Sharif Razavian et al. (2015, 2019). Furthermore, we have shown that muscle synergies can be used to further simplify the control process. Here the basics of the proposed motor control are briefly described.

**Figure 2:**
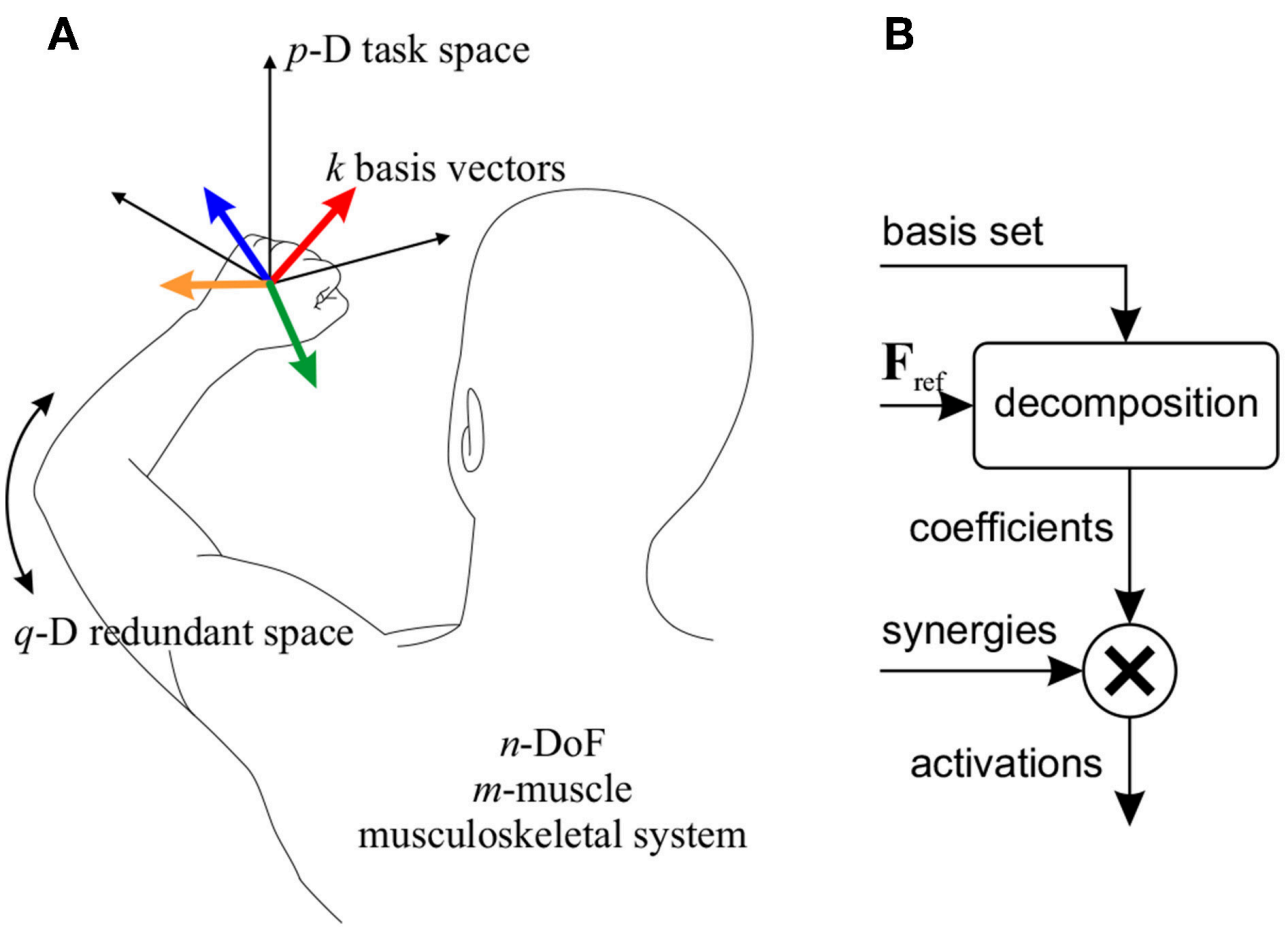
**(A)** Definition of the parameters used in the motor control framework. In the example shown, a 3D task space (*p* = 3) for a reaching task is considered. *k* = 4 basis vectors (colored vectors) are shown here corresponding to the same number of synergies. In this example, the remaining degree of freedom forms the redundant space (*q* = 1). **(B)** The block diagram of the motor control framework. A reference force vector (**F**_ref_) is decomposed onto the basis set, and the coefficients are used to combine the corresponding the synergies.

Assume an *n*-DoF musculoskeletal system with *m* muscles (*m*>*n*, Figure 2A), for which a *p*-dimensional task space is considered (*p* ≤ *n*). To control the motion in this task space, *k* synergies are defined. In this context, a “synergy" is defined as the activation of a group of muscles with predetermined relative ratios, and is expressed as a column-vector *S*_*m*×1_ containing the activation ratios. The synergy matrix, **S**_*m*×*k*_, is formed by concatenating the *k* synergy vectors. It is further assumed that the relative activation ratios may change based on the posture (de Rugy et al., 2012; Sharif Razavian et al., 2015); therefore, the synergies are *posture-dependent*.

In this motor control framework, it is also assumed that each synergy produces a *p*-D force vector in the task space; the collection of all synergy-produced vectors can be viewed as a basis set for the *p*-D task space (i.e., any arbitrary task space force vector can be written as a linear combination of these basis vectors). Therefore, corresponding with the synergies, there is the basis set **B**_*p*×*k*_.

If the needed body motion in the task space is known (which may be defined by a high-level task space controller), the required task space force vector can be decomposed onto the basis set, to find the coefficient of each basis vector. Then, combining the synergies with the corresponding coefficients will result in muscle activations that move the body in the desired direction in the task space (Figure 2B).

By assuming that the basis vectors have no component in the redundant space, it is possible to control the task variables and leave the redundant ones uncontrolled. In other words, under this assumption, the muscle activities do not induce any extra force in the redundant space, which implies that these degrees of freedom are not controlled by the muscles. However, there are situations where the redundant DoFs are important and need to be actively controlled (for example to avoid an obstacle or reach a target at a certain angle). Therefore, the task space in these situations includes all the degrees of freedom.

Our motor control framework requires synergies that are defined for a specific task space. Thus, a new set of synergies would ideally be required for the new full-dimensional task space. However, increasing the dimensions of the problem exponentially increases its complexity. Therefore, we have made an assumption to avoid such complexities.

Instead of defining new synergies for the new *n*-D task space, we have taken the original ones (**S**_*m*×*k*_) and augmented it with *l* new synergies (${\bar{\text{S}}}_{m\times l}$) that produce basis vectors orthogonal to the original ones. Mathematically, the augmented synergy matrix $\hat{\text{S}}$ is written as:

(1) $$\begin{array}{l} {\hat{\text{S}}}_{m\times \left(k+l\right)}=\left[{\text{S}}_{m\times k}\;{\bar{\text{S}}}_{m\times l}\right] \end{array}$$

which produces the basis set:

(2) $$\begin{array}{l} {\hat{\text{B}}}_{n\times \left(k+l\right)}=\left[\begin{array}{cc} {\text{B}}_{p\times k} & 0\\ 0 & {\bar{\text{B}}}_{q\times l} \end{array}\right] \end{array}$$

Here, $\hat{\text{B}}$ is the augmented basis set for new *n*-D task space, and ${\bar{\text{B}}}_{q\times l}$ is the basis set that spans the original redundant space (*q* is the dimensions of the original redundant space, *p* + *q* = *n*). Therefore, the *n*-D task space is spanned by the collection of two basis sets that are orthogonal to each other. To put this in the motor control framework, the high-level controller may now define an *n*-D reference force vector ${\hat{\text{F}}}_{n\times 1}$, which is then decomposed into the basis set:

(3) $$\begin{array}{l} {\hat{\text{F}}}_{n\times 1}={\left[\begin{array}{c} {\text{F}}_{p\times 1}\\ {\bar{\text{F}}}_{q\times 1} \end{array}\right]}_{ref}={\hat{\text{B}}}_{n\times \left(k+l\right)}{\hat{\text{C}}}_{\left(k+l\right)\times 1} \end{array}$$

to find the coefficients of the synergies, $\hat{\text{C}}$. These coefficients are then used as the weightings to combine the synergies as:

(4) $$\begin{array}{l} {\text{u}}_{m\times 1}={\hat{\text{S}}}_{m\times \left(k+l\right)}{\hat{\text{C}}}_{\left(k+l\right)\times 1} \end{array}$$

The resulting muscle activations, **u**_*m*×1_, will produce the force vectors **F**_*ref*_ and ${\bar{\text{F}}}_{ref}$ in the original task and redundant spaces, respectively.

This formulation, although being sub-optimal compared to a general *n*-D basis set, has the advantage of decoupling the task and redundant spaces. As a result, it is possible to switch on/off the control of the redundant DoFs by choice. The task space controller may output a non-zero reference force in the redundant space (${\bar{\text{F}}}_{ref}\neq 0$) to control it, or a zero value (${\bar{\text{F}}}_{ref}=0$) to leave it uncontrolled. Because of the architecture of the motor control framework, the condition ${\bar{\text{F}}}_{ref}=0$ does not enforce zero movement—it means no extra force is produced by the muscles. Another interesting implication of this method is the possibility of implementing a less strict controller, for a loose control of the redundant DoFs.

## 3. Human Experiments

We have performed human motion analysis to evaluate the validity of the assumptions. Specifically, the goal was to investigate how well the assumption of the task/redundant space orthogonality works in practice to estimate the muscle activities from task/redundant space measurements.

**Figure 3:**
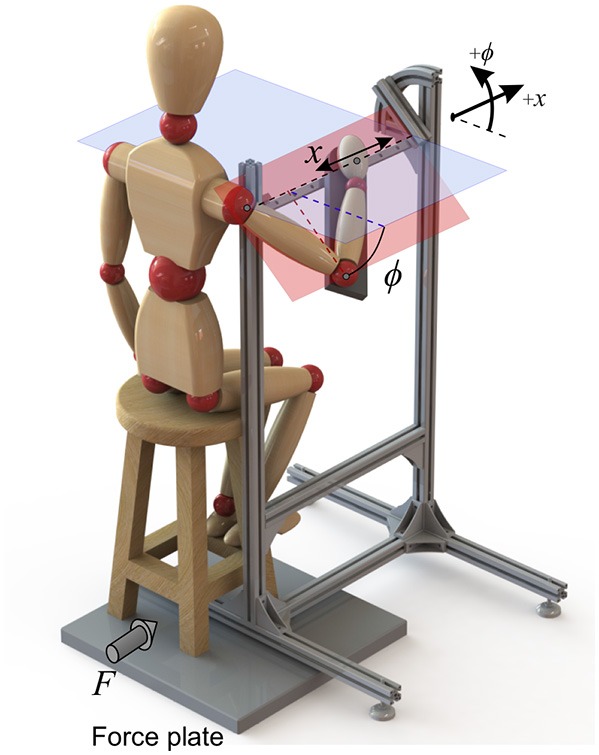
The experimental setup that restricts the arm motion to only two DoFs. Force plates measure the ground reactions, and the kinematics are collected by an optical motion capture system.

For this purpose, the experimental set-up of Figure 3 was designed to impose certain constraints on the body. The subject was asked to hold the handle, and the forearm was strapped to the armrest. As a result, the set-up allowed only two degrees of freedom: a linear motion of the hand in the *x* direction (which is considered as the *task space*), plus a rotation of the arm about the same axis (the *redundant space*, denoted by angle *ϕ*). These two DoFs and their positive directions are shown in Figure 3.

Surface electromyogram (EMG) data from eight muscles (Table 1) was recorded at 1926 Hz (Trigno Wireless EMG, Delsys Inc.). The arm motion was recorded at 150 Hz (Optotrak Certus, Northern Digital Inc.), with optical markers placed on the shoulder (acromion process), elbow (lateral epicondyle), wrist (ulnar styloid process) and hand (distal end of the third metacarpal bone). Force plate data was also recorded at 100 Hz (AccuGait, Advanced Mechanical Technology, Inc.). By assuming negligible body motion, the forces at the hand can be sensed by the force plate beneath the stool.

**Table 1:** The muscles recorded in the experiments.

**# Muscle**	**# Muscle**
1 Anterior deltoid	5 Brachioradialis
2 Middle deltoid	6 Long head of triceps brachii
3 Posterior deltoid	7 Lateral head of triceps brachii
4 Biceps brachii	8 Pectoralis major

The EMG data were processed with the common procedure (raw EMG → zero-mean → band-pass filter with 6th-order Butterwork and 5–800 Hz cut-off frequency → full-wave rectify → low-pass filter with 6th-order Butterworth and 2 Hz cut-off frequency). Since the tested movements did not involve fast dynamics, a 2 Hz cut-off frequency was chosen for the low-pass (linear envelope) filter to produce smooth signals. The bandpass filter cut-off frequencies were chosen to remove the biases in the signals while retaining as much information as possible. The EMGs for each muscle were normalized with respect to the 95th percentile of the observed signal during the entire experiment.

Six subjects (two female, four male, average age 26.3 ± 3.0, all right-handed, no prior musculoskeletal disorder/injury) participated in the study. The experiment was approved by the Office of Research Ethics at the University of Waterloo.

The experiments were conducted in two phases. The goal of the first phase was to obtain the posture-dependent orthogonal task/redundant muscle synergies, which were used in the second phase (movement trials) to estimate the muscle activities from the kinematic/dynamic measurements.

### 3.1. Phase One: Obtaining the Synergies

First, the subject was asked to exert positive isometric forces (push) along the task space. The set-up was locked at 9 different positions (three positions along the task space {close, middle, far}, each at three angles in the redundant space {0°, −45°, −90°}). Therefore, a total of nine 5-s sessions were recorded. At each posture, the average EMG data from the eight muscles was divided by the average pushing force, ${\bar{F}}^{+}$, to obtain the “synergies” that produce unit force in the task spaces; i.e.,:

(5) $$\begin{array}{l} \text{positive task space synergy}=\frac{{\left[\bar{\text{EMG}}\right]}_{8\times 1}}{{\bar{F}}^{+}} \end{array}$$

Next, 8 second-order polynomial surfaces [*f*_*i*_(*x*, *ϕ*), *i* = 1…8, one for each muscle] were fitted to these *posture-dependent* synergy data, which allowed estimation of the synergy vector in any given posture.

(6) $$\begin{array}{l} {\text{S}}_{task}^{+}\left(x,\phi \right)=\left[\begin{array}{c} f_{1}\left(x,\phi \right)\\ f_{2}\left(x,\phi \right)\\ \vdots \\ f_{8}\left(x,\phi \right) \end{array}\right] \end{array}$$

The same procedure was repeated in the negative direction of the task space (the subject was instructed to *pull* along the task space), which resulted in negative task space synergy ${\text{S}}_{task}^{-}\left(x,\phi \right)$. Additionally, rotations in the positive and negative directions in the redundant space were also tested to obtain the redundant synergies ${\text{S}}_{red}^{+}\left(x,\phi \right)$ and ${\text{S}}_{red}^{-}\left(x,\phi \right)$. To calculate these functions, the posture-dependent synergy data were obtained by dividing the EMGs by the measured redundant space torque:

(7) $$\begin{array}{l} \text{positive redundant space synergy}=\frac{{\left[\bar{\text{EMG}}\right]}_{8\times 1}}{{\bar{T}}^{+}} \end{array}$$

(8) $$\begin{array}{l} \text{negative redundant space synergy}=\frac{{\left[\bar{\text{E}M\text{G}}\right]}_{8\times 1}}{-{\bar{T}}^{-}} \end{array}$$

As no training effect was expected, the order of conducting the trials was fixed and as follows: (1) positive task, *F*^+^; (2) negative task, *F*^−^; (3) positive redundant, *T*^+^; (4) negative redundant, *T*^−^. The subject finished the isometric tests in all required postures before moving to the next force or torque direction.

By concatenating the calculated synergy vectors, the synergy matrix and the corresponding basis vectors can be constructed as:

(9) $$\begin{array}{l} {\hat{\text{S}}}_{8\times 4}\left(x,\phi \right)=\left[{\text{S}}_{task}^{+}\;{\text{S}}_{task}^{-}\;{\text{S}}_{red}^{+}\;{\text{S}}_{red}^{-}\right] \end{array}$$

(10) $$\begin{array}{l} {\hat{\text{B}}}_{2\times 4}=\left[\begin{array}{cccc} +1 & -1 & 0 & 0\\ 0 & 0 & +1 & -1 \end{array}\right]\begin{array}{c} \leftarrow \text{task dimension}\\ \leftarrow \text{redundant dimension} \end{array} \end{array}$$

Here, the basis vectors are the unit force/torque in the combined 2D space of (*x*, *ϕ*).

Note that this definition of muscle synergies is different from what is generally used in the literature. The usual practice is to collect EMGs in various conditions, and use a dimension-reduction algorithm (e.g., non-negative matrix factorization or principal component analysis) to extract the synergies (Tresch et al., 2006). In our approach, we have assumed that the directly measured co-activation of the muscles is a synergy by itself (no dimension-reduction required). The traditional methods need significantly more EMG data in each posture for reliable factorization and provide little control over the directions of the basis vectors (i.e., to impose orthogonality).

### 3.2. Phase Two: Motion Trials

In the second phase, the subject was instructed to reach forward and backward along the slider; therefore, the instructed *task* was a point-to-point reach. There was a small resistance on the slider to increase the muscular activity, and the subject performed the movement for 120 s at a self-selected speed. The redundant DoF was once fixed (locked at three different angles {0°, −45°, −90°}), and once left free to rotate. In the free motion trials, the subject was asked to actively hold the arm at specific angles {0°, −45°, −90°} while reaching forward and back. Lastly, the subject was asked to disregard the redundant angle, and *naturally* move back and forth in the task space. The three sets of trials are designated by *fixed, controlled*, and *natural* trials, respectively, and are summarized in Table 2.

**Table 2:** The motion trials in the experiments.

**Trial name**	**Redundant DoF**	**Trial name**	**Redundant DoF**
*Fixed-0*	Fixed at 0°	*Controlled-0*	Free, held at 0°
*Fixed-45*	Fixed at −45°	*Controlled-45*	Free, held at −45°
*Fixed-90*	Fixed at −90°	*Controlled-90*	Free, held at −90°
		*Natural*	Free, unattended

In these trials, it was assumed that a task space controller (unknown nature at this moment) had decided on the movement trajectories that satisfied the tasks mentioned above. The task/redundant space forces are assumed to be the measurable outputs of this high-level controller. The goal is to use these observed task/redundant space forces to estimate the muscle activations. Therefore, the procedure introduced in section 2 is used to reconstruct the muscle activities.

At any time step [*t*] during the motion, the coefficients vector, ${\hat{\text{C}}}_{4\times 1}={\left[c_{task}^{+},c_{task}^{-},c_{red}^{+},c_{red}^{-}\right]}^{\text{T}}$, was calculated such that:

(11) $$\begin{array}{l} {\left[\begin{array}{c} F\left[t\right]\\ T\left[t\right] \end{array}\right]}_{measured}={\hat{\text{B}}}_{2\times 4}{\hat{\text{C}}}_{4\times 1} \end{array}$$

where ${\hat{\text{B}}}_{2\times 4}$ is defined in Equation (10). The calculated coefficients are then multiplied by the synergy matrix:

(12) $$\begin{array}{l} {\text{EMG}}_{est}\left[t\right]=\hat{\text{S}}{\left(x\left[t\right],\phi \left[t\right]\right)}_{8\times 4}{\hat{\text{C}}}_{4\times 1} \end{array}$$

to estimate the muscle activations. To quantify this estimation performance for the *i*th muscle, the *variance accounted for* (VAF, Roh et al., 2012; de Rugy et al., 2013) is used:

(13) $$\begin{array}{l} VAF_{i}=1-\frac{\sum_{t}{\left({\text{EMG}}_{i}\left[t\right]-{\text{EMG}}_{est,i}\left[t\right]\right)}^{2}}{\sum_{t}{\text{EMG}}_{i}{\left[t\right]}^{2}} \end{array}$$

where the summation is over all the time steps during the movement.

**Note 1:** Since the basis vectors in Equation (10) are orthonormal (they are unit vectors orthogonal to each other), the decomposition in Equation (11) is essentially a separation of positive and negative portions of the measured force/torque.

**Note 2:** The synergy matrix $\hat{\text{S}}\left(x\left[t\right],\phi \left[t\right]\right)$ in Equation (12) is the posture-specific synergy matrix, calculated using the fitted polynomial surfaces Equation (6).

**Note 3:** We have assumed that the redundant DoF control was turned off in the *fixed* trials. Therefore, zero redundant space torque (*T* = 0) was used in Equation (11) to reconstruct the muscle activations. In other trials, the redundant space torque could not be measured with our apparatus; thus it was calculated as:

(14) $$\begin{array}{l} T=T_{max}\cos\left(\phi \right)\frac{d}{d_{max}} \end{array}$$

where *T*_*max*_ is the torque due to the weight of the arm when *ϕ* = 0° and the elbow is fully flexed, and is measured using counter weights in a static pose. When the arm is fully flexed, the distance of the elbow from the axis of rotation is *d*_*max*_, which is used to scale the measure distance *d* during the movements.

### 3.3. Statistical Analyses

Due to the specificity of the recordings to the individual subjects and measurement conditions, direct inter-subject comparison of the recorded signals was not practical. Therefore, to make statistical arguments, the calculated VAF (as the quantitative similarity measure between the measured and estimated muscle activations) was used. The Kruskal-Wallis test was used to test whether the distribution of the results was different across subjects, test scenarios, or muscles.

The results of the orthogonality condition were compared against other methodologies. Specifically, to compare the estimation performance of the proposed method to a baseline, a random (by-chance) estimator was constructed, which matched the mean and standard deviation of the measured muscle activities. Additionally, to evaluate the significance of the orthogonal synergies, the framework was re-applied to the movement data with two alterations: (1) with the redundant synergies turned off, and (2) with double the effect [coefficients $c_{red}^{+}$ and $c_{red}^{-}$ multiplied by a factor of two in Equation (12)]. These two conditions do not change the results for the *fixed* trials, and have effect only on *uncontrolled* and *natural* trials.

## 4. Results

Six subjects participated in the experimental trials. In the following, the results belonging to subject #1 are shown. The detailed results for all subjects are provided in the Appendix.

### 4.1. Posture-Dependent Synergies

**Figure 4:**
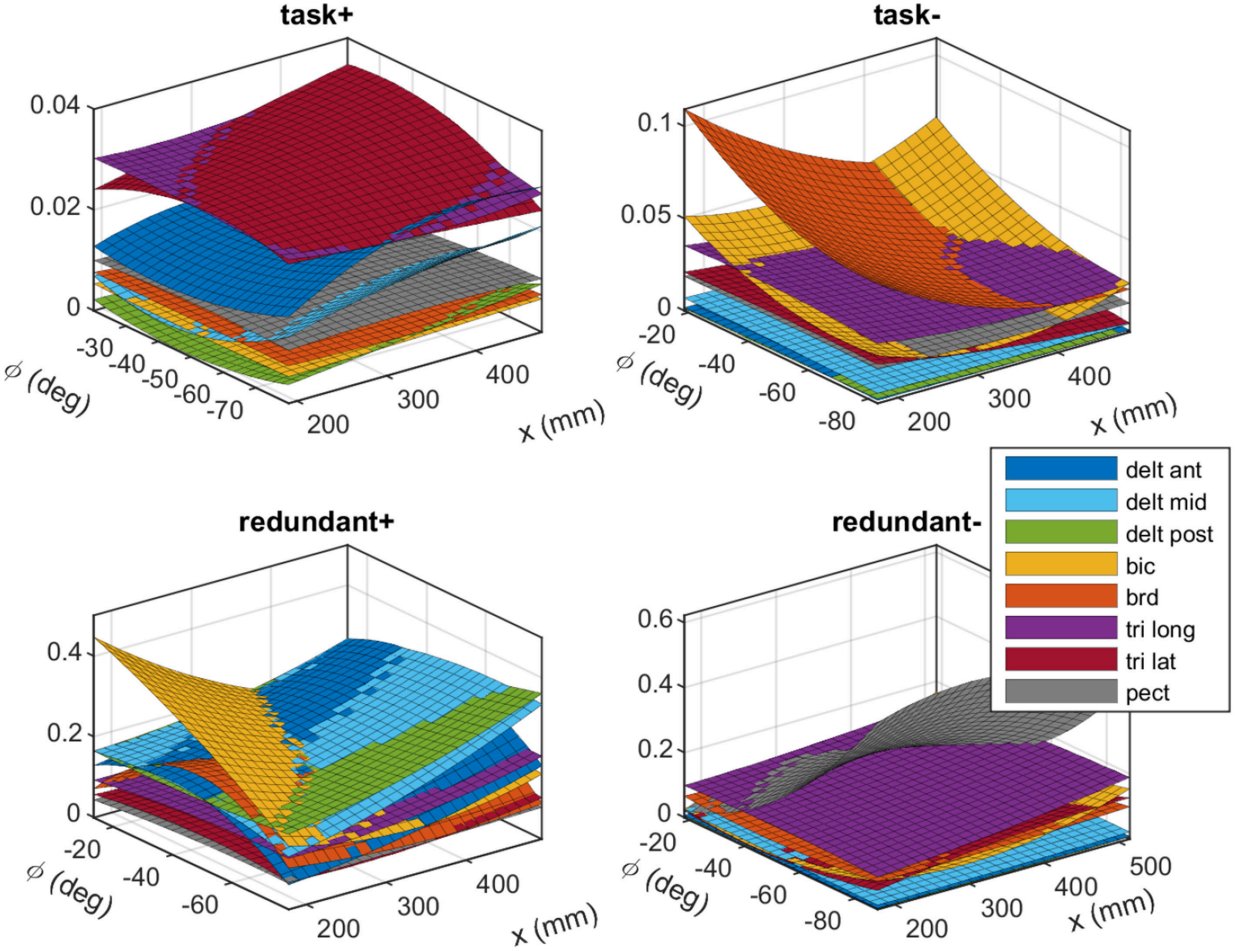
The experimentally obtained synergies that correspond to the task space (top row) and the redundant space (bottom row). The plots show fitted polynomial functions to the measured data. Each surface (color coded) show how a muscle's activation in a synergy changes across various postures. Each synergy produces unit force (or torque) in the task space (or redundant space). These synergies belong to subject #1. Detailed plots are available in the Appendix.

The experimentally obtained synergies from subject #1 are shown in Figure 4. These plots show the fitted polynomial surfaces for each muscle in different synergies, and how the activity of the muscles in the synergies changes by posture. At a given posture (*x*, *ϕ*) the value of each surface corresponds to the elements of the synergy matrix **S**. The plots “task+” and “task−” show the task space synergies (pushing and pulling along the linear guide, respectively), while the “redundant+” and “redundant−” plots show the redundant space synergies (roughly translate into elevating the arm and lowering it, respectively).

### 4.2. Motion Trials

**Figure 5:**
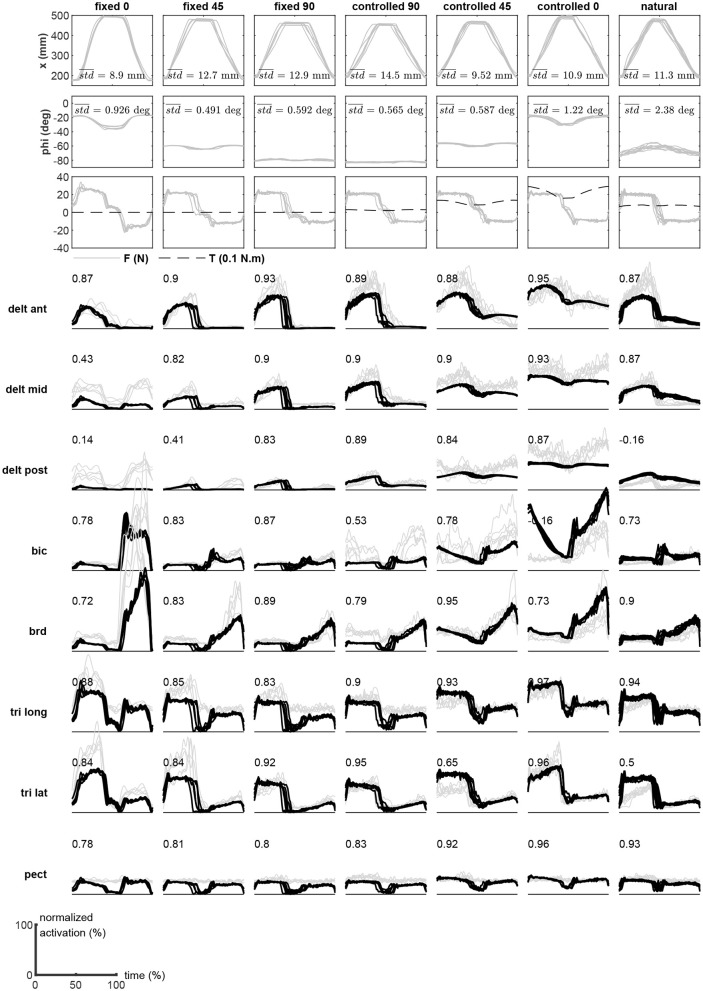
The experimental results (subject #1) during the motion trials. The measured motion in the task and redundant spaces, measured forces, and the recorded EMGs are shown. The EMGs (gray lines) are overlaid with the reconstructed activities (black) during the motion trials.

Subject #1's recorded motion in the task and redundant spaces are shown in the top two rows of Figure 5. These plots show nine repetitions of the motion (reach out, rest, and return). Overlaid on each plot is the average variation in the trial, which is calculated as the mean of the point-by-point standard deviations during the movement. The third row shows the measured task space force, as well as the estimated redundant space torque. Finally, the comparison of the measured and estimated muscle activities during movements are shown in the subsequent rows of Figure 5. To obtain these results, the measured *x* and *ϕ* values at a given time are used to calculate the synergies from the surfaces shown in Figure 4. Next, the obtained synergy matrix along with the measured force at this time is used in Equations (11) and (12). This process is performed for the entire duration of the movement. The average computation time in each time step is 231 μs (Intel Core i7-6700 CPU and 16 GB RAM, running Matlab 2018b).

The VAF Equation (13) has been used to quantify the estimation performance of the method. The numbers presented in the plots of Figure 5 are the VAF calculated for the individual muscles during the specific trial.

To compare the overall estimation performance of the method across the subjects, the overall VAF (weighted mean of VAF over all muscles/scenarios) is used. These numbers are reported in Table 3 and are calculated as:

(15) $$\begin{array}{l} {VAF}_{overall}=\frac{\sum \left(\bar{EMG_{i}}.VAF_{i}\right)}{\sum \bar{EMG_{i}}} \end{array}$$

where $\bar{EMG_{i}}$ is the average value of the recorded EMG for the *i*th muscle and during a test scenario. The summations in Equation (15) are taken across all muscles and scenarios. The mean of the overall VAF across all subjects is 0.695 with a standard deviation of 0.098.

**Table 3:** The overall VAF calculated for individual subjects.

**Subject #**	**VAF**	**Subject #**	**VAF**
1	0.826	4	0.772
2	0.694	5	0.575
3	0.595	6	0.709
Mean ± SD = 0.695 ± 0.098			

### 4.3. Statistical Analysis

**Figure 6:**
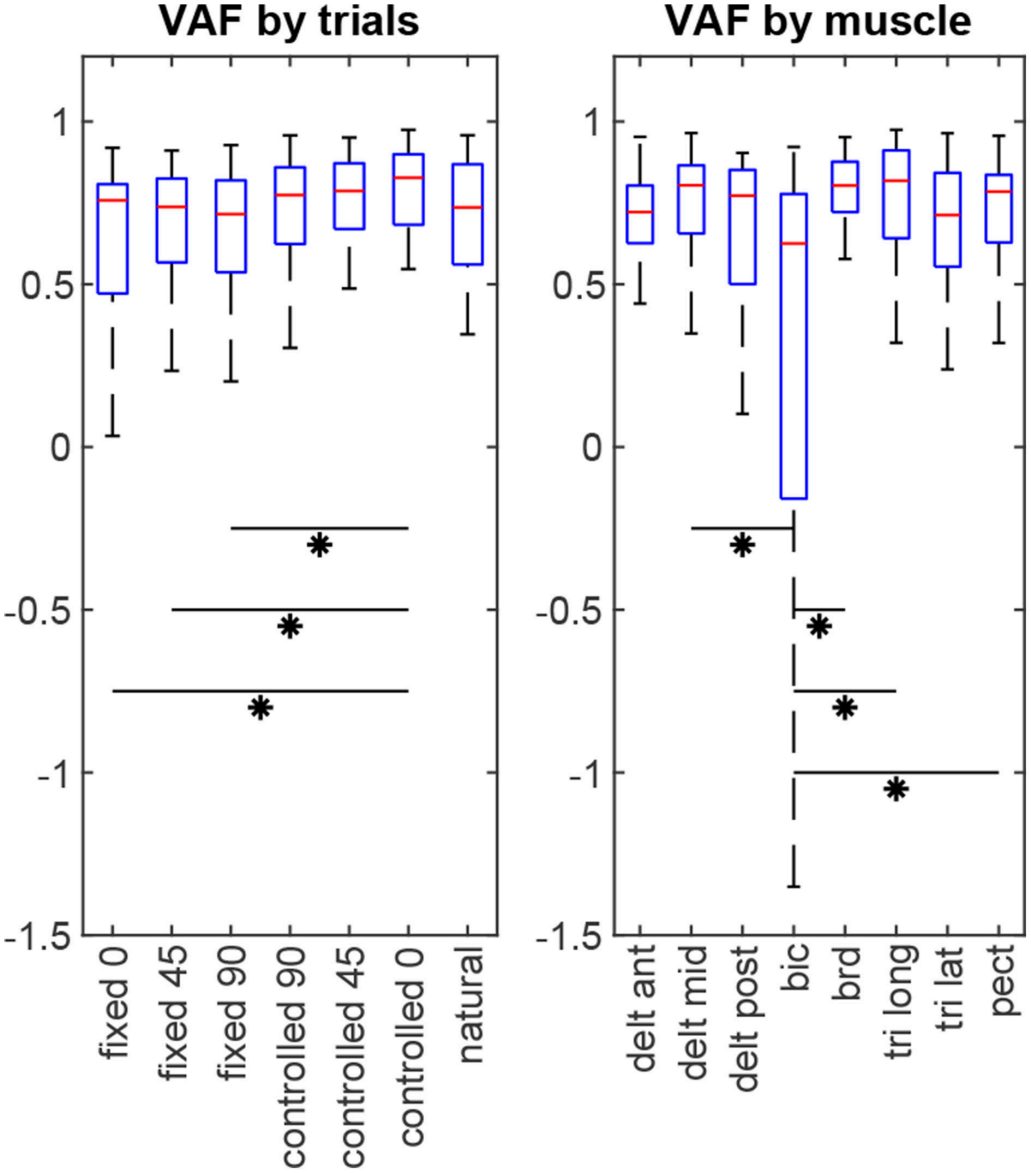
The estimation performance (VAF) comparison by trials and by muscles. Asterisks indicate statistical significance.

For each subject, 8 × 7 = 56 VAF values are calculated (8 muscles in 7 test scenarios). The non-parametric Kruskal-Wallis test shows that there is no statistical difference between subjects' results (*p* = 0.32). Considering all subjects' data together, statistical analysis (Figure 6) reveals that the estimation performance for the *controlled-0* trial is better than the fixed trials (*p* = 0.026, 0.034, 0.020 for *fixed-0*, –*45* and –*90*, respectively), but not different from the rest. Furthermore, the estimation quality for biceps is significantly lower than those for middle deltoid (*p* = 0.008), brachioradialis (*p* < 10^−5^), long head of triceps (*p* = 10^−4^), and pectoralis major (*p* = 0.023). All other muscle pairs have insignificant VAF differences (*p* >0.05).

The baseline from the by-chance estimator is calculated to be VAF = 0.467 ± 0.126 (inter-subject mean ± standard deviation), which is statistically inferior to the results of the synergy-based method (*p* < 10^−5^). Additionally, including the orthogonal synergies reconstructs the muscle activities better than ignoring them (overall VAF = 0.597 ± 0.233, *p* < 10^−5^), or using them with double the effect (overall VAF = 0.329 ± 1.66, *p* = 0.011). A noteworthy observation is that the estimation results from the *natural* trials change slightly when turning the redundant space control on or off. Although using the orthogonal synergies improves the overall estimation for this trial across all subjects (turned on: VAF = 0.619 ± 0.474; turned off: VAF = 0.590 ± 0.232, *p* = 0.044), it introduces significantly more variability into the estimation performance.

**Figure 7:**
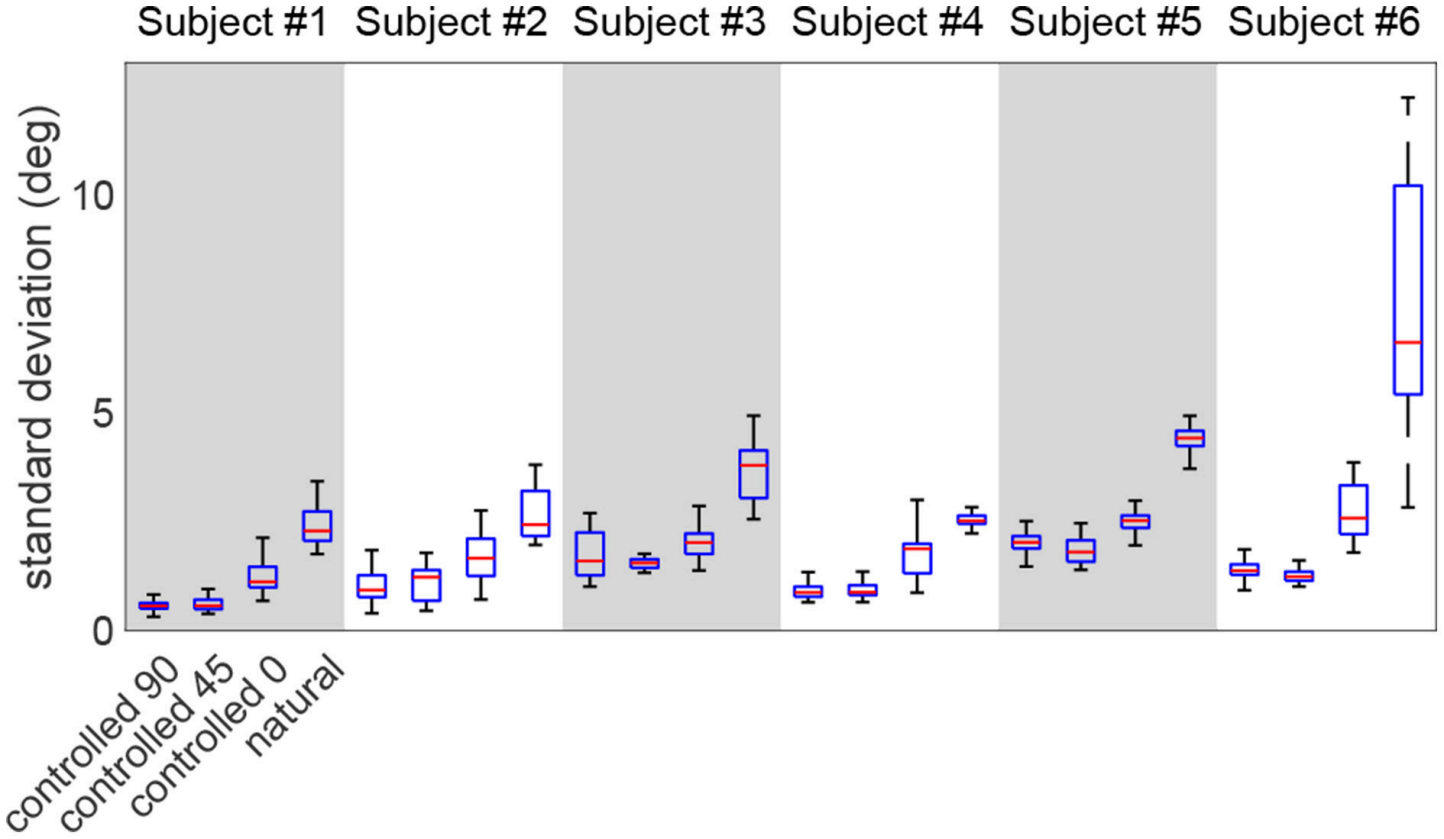
The comparison of the standard deviation distribution of the redundant DoF during various test scenarios. The *natural* trial exhibits the largest standard deviations.

Figure 7 shows the distribution of the point-by-point standard deviations across multiple repetitions, in various test scenarios (the mean value is the one reported in Figure 5). This figure compares the standard deviations between the *natural* and *controlled* scenarios. As expected, the *natural* trial exhibits larger standard deviations than *controlled* tests in all subjects (*p* < 10^−5^). Moreover, the *controlled-0* trial also shows significantly larger variation than *controlled-90* and *controlled-45* in all subjects (*p* < 3 × 10^−4^). The *controlled-45* and *controlled-0* are not different, except for subjects #5 and #6 (*p* < 2 × 10^−2^).

## 5. Discussion

The advantages of task space representation in motor control were previously shown in Sharif Razavian et al. (2015, 2019), where explicit synergy/task relations were used to estimate the muscle activities that satisfied task requirements. Although this framework was successful in real-time control of the motion in the task space, the redundant degrees of freedom were essentially left uncontrolled. Similar studies that considered task/synergy relationships (e.g., D'Avella et al., 2008; Berger and D'Avella, 2014; Fu et al., 2015) also lack discussion of the redundant DoFs. The present paper introduces an extension to the motor control framework of Sharif Razavian et al. (2019) by proposing how the same framework can be used to control the redundant degrees of freedom alongside the task-related ones.

The main assumption made here was the orthogonality of the basis sets in the task and redundant space. This assumption stems from the observations that the nervous system controls the task-related variables more tightly than the task-irrelevant ones (see the uncontrolled manifold theory, Scholz and Schöner, 1999). This separation of control suggests that the actuations affecting the task variables do not strongly affect the redundant ones; thus, the actuator actions in the task space must be orthogonal to the redundant spaces. Although our experimental results cannot be used as a proof for the existence of orthogonal synergies within the human nervous system, they showed that, at least at the behavioral level (i.e., EMG), the orthogonality assumption yielded acceptable estimation performance; about 70% of the muscle activation variations can be reconstructed using the assumption of orthogonal bases for the task/redundant spaces. As a practical conclusion, the control of motion in the task and redundant spaces can be separated in a computational model by employing orthogonal basis vectors, and the results are not far from reality. Therefore, we can build mathematical motor control models more confidently using this orthogonality assumption, which are especially useful for the real-time model-based control of bio-mechatronic systems (Mehrabi and McPhee, 2019), rehabilitation robots (Ghannadi et al., 2018), exoskeletons (Kuhn et al., 2018), and functional electrical systems (Sharif Razavian et al., 2017, 2018).

The orthogonality condition simplifies the computations in a feedback motor control model, as the task-related synergies will not induce motion in the redundant space, and vice versa. This assumption also introduces the ability to switch on/off the redundant space control, without the need to change the control structure. Furthermore, it is possible to construct separate feedback loops with different tracking properties to control the task and redundant degrees of freedom (e.g., a tight control of task variables, and a less strict controller for the redundant ones).

The downside to using the orthogonal synergies, however, is the sub-optimality of the calculated muscle activities. The “gold standard” computational tool to estimate muscle activities in the literature is optimization, which is computationally intensive and requires significantly more information (e.g., full kinematic measurements, body segment mass properties, and muscle parameters). Our motor control model is easier to compute and relies on less information (only positions/forces in the task and redundant space). To improve the results, it is possible that a general (non-orthogonal) *n*-D basis set would result in more efficient muscle activations. Reflecting upon the physiological aspects of this issue, one idea is similar to that presented by Raphael et al. (2010) and Loeb (2012): it might be possible that in a novel situation, the nervous system starts employing the previously known solutions (a basis set for the task space, plus another one orthogonal to the first for the redundant space), and eventually explores the neighboring solutions until it reaches a “good-enough” solution (a non-orthogonal basis set, with possibly fewer synergies).

Note that the definition of the synergies in our experiments was different from the mainstream in the literature. Although our synergies comply with the concept of “coordinated recruitment of a group of muscles with specific amplitude balances” (D'Avella, 2016), they are not obtained through a dimension reduction procedure (e.g., non-negative matrix factorization, Tresch et al., 2006). Instead, we took the recorded activity of the muscles for a certain force direction, and directly defined them as a synergy. The reason for this approach is that the considered task/redundant spaces are both one-dimensional, and therefore, only two synergies are observable from the measured data (one in each direction of movement along the task or redundant space). It is the same approach suggested by Sharif Razavian et al. (2015) that only two synergies suffice to fully and optimally control a one-DoF task space.

Another contrasting point between our synergies and the literature is their posture-dependence definition. This added flexibility in the synergies is a key feature that allows explaining more muscle activity variation with a smaller number of synergies. For maximum control performance, the synergies can also be velocity-dependent (D'Avella et al., 2008). However, the added benefit of velocity dependence is small compared to that of the posture-dependence (Sharif Razavian et al., 2015).

Our results show that switching off the redundant space controller significantly degrades the estimation performance (overall VAF across all subjects drops from 0.695 to 0.597, *p* < 10^−5^); however, this effect on the *natural* trials is less profound (VAF changes from 0.619 to 0.590, *p* = 0.044). These results indicate that the redundant controller is essential for a better estimation in the *controlled* trials, but is not as important in the *natural* trial. This suggests a less active control of the redundant DoFs by the nervous system. Furthermore, the larger variation of the VAF across subjects during the *natural* trial (VAF standard deviation = 0.474 and 0.232, for redundant synergies turned on and off, respectively) might be due to the subject-specific differences in the level of engagement of redundant space controller.

Although the estimation of the muscle activities was not significantly different between subjects and most trials/muscles, a few differences were observed. The biceps was the only muscle that showed a different distribution (and had lower estimation quality), which could be attributed to poor EMG collection conditions. The biceps muscle belly moves significantly under the skin-mounted electrode, which degrades the quality of EMG signals. Another observation is the higher estimation performance for the *controlled-0* trial (compared to *fixed* scenarios, *p* < 0.034). We speculate that, since this trial is the most demanding one (holding the arm at an elevated position against gravity while moving back and forth), muscles are the most active, resulting in higher signal-to-noise ratio and better estimations.

The experimental results were obtained following an *inverse* approach; i.e., the muscle activations were estimated from the measured kinematics/dynamics of the arm. In the context of a motor control model, considering a *forward* approach (estimate the motion from the muscle activation commands) is also important, especially when predictive control is intended (Berger and D'Avella, 2014). In general, the muscle activation estimated from an inverse approach will not result in the same desired motion, mostly due to estimation errors, disturbance/noise, and unknown dynamics. However, by putting this inverse mapping in a hierarchical feedback control scheme, it is possible to compensate for much of the error, and achieve acceptable control performance (Sharif Razavian, 2017; Sharif Razavian et al., 2018; Sharif Razavian et al., 2019.

Lastly, the calculated motion variation in the redundant space (*ϕ* trajectories in Figure 5, further detailed in Figure 7) is expectedly higher in the *natural* trial in all subjects compared to other test scenarios. This observation is in agreement with the notion of an uncontrolled manifold (Scholz and Schöner, 1999), as the arm angle is actively kept constant during *controlled* trials. This observation suggests that *ϕ* is controlled less tightly by the nervous system during natural reaching tasks. Additionally, it was noted that the *controlled-0* trial exhibits higher variation than *controlled-0/45*, which is expected given that elevating the arm is a more demanding task.

## 6. Conclusions

A motor control framework for fast feedback control of complex musculoskeletal systems was previously presented (Sharif Razavian et al., 2019), which was based on the relationship between muscle synergies and the task space. In this paper, the idea of task space control was extended by introducing orthogonal basis sets in the task and redundant spaces. As a result, the motor control framework is now capable of handling kinematic redundancies, with the option to selectively switch off their control (leave them uncontrolled). We performed an experimental trial to examine how well this computational model (with orthogonal synergies) can estimate muscle activities from task/redundant space measurements. The experimental data showed that the assumption of orthogonal task/redundant bases can estimate the muscle activities from the measured kinematics/dynamics in the task and redundant spaces with approximately 70% accuracy. These observations build confidence in using this motor control framework with orthogonal bases in computational models and control applications, as a fast alternative to optimization-based methods.

## Ethics Statement

The experiment was approved by the Office of Research Ethics at the University of Waterloo. All subjects gave written consent to participate in this study.

## Author Contributions

All authors listed have made a substantial, direct and intellectual contribution to the work, and approved it for publication.

### Conflict of Interest Statement

The authors declare that the research was conducted in the absence of any commercial or financial relationships that could be construed as a potential conflict of interest.
