# Supplementary material for: On the Relationship Between Muscle Synergies and Redundant Degrees of Freedom in Musculoskeletal Systems
Source: Front Comput Neurosci. 2019 Apr 16;13:23. doi: 10.3389/fncom.2019.00023 (PMC6477041; doi:10.3389/fncom.2019.00023)
Supplement: Supplementary file 1 [file Data_Sheet_1.pdf]

Sharif Razavian R, Ghannadi B and McPhee J (2019) "On the Relationship Between Muscle Synergies and Redundant Degrees of Freedom in Musculoskeletal Systems". Frontiers in Computational Neuroscience 13:23. doi: 10.3389/fncom.2019.00023

APPENDIX: Extended Results

This appendix presents the detailed experimental results for all six subjects. Figures 1 through 6 show how individual muscle activations change with posture in different synergies. Figures 7 through 12 show the motion trial results. For detailed description of the figures, please refer the original text.

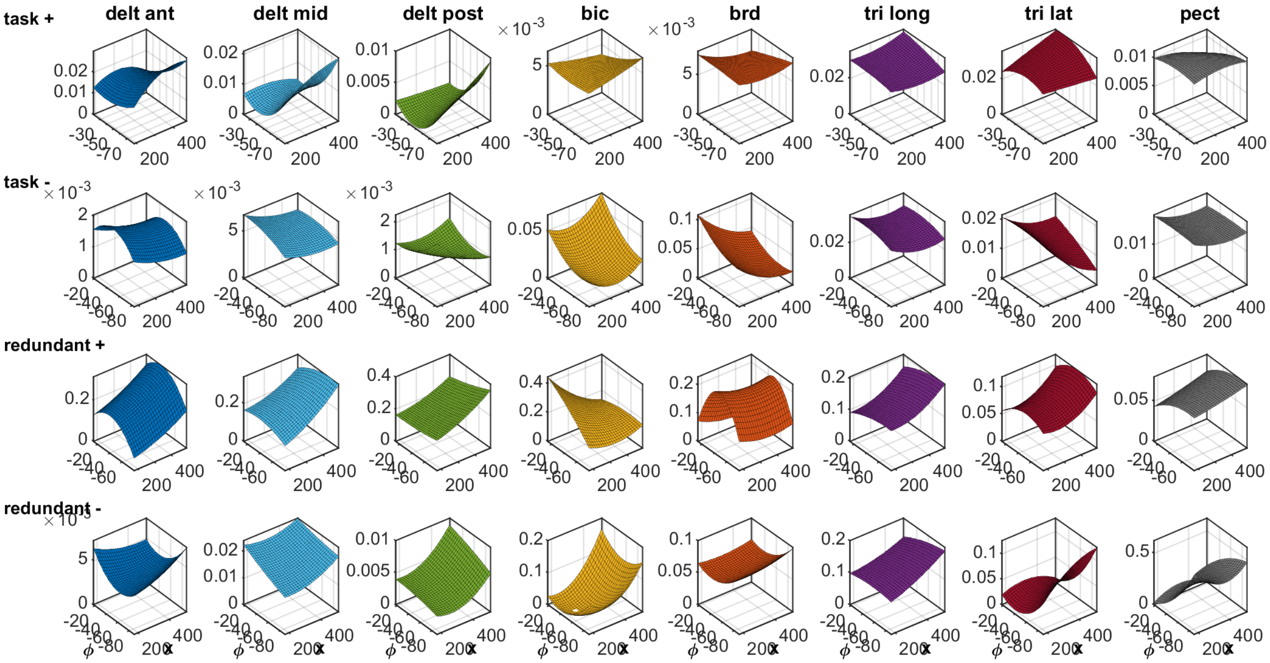

Figure 1: Subject #1's synergies.

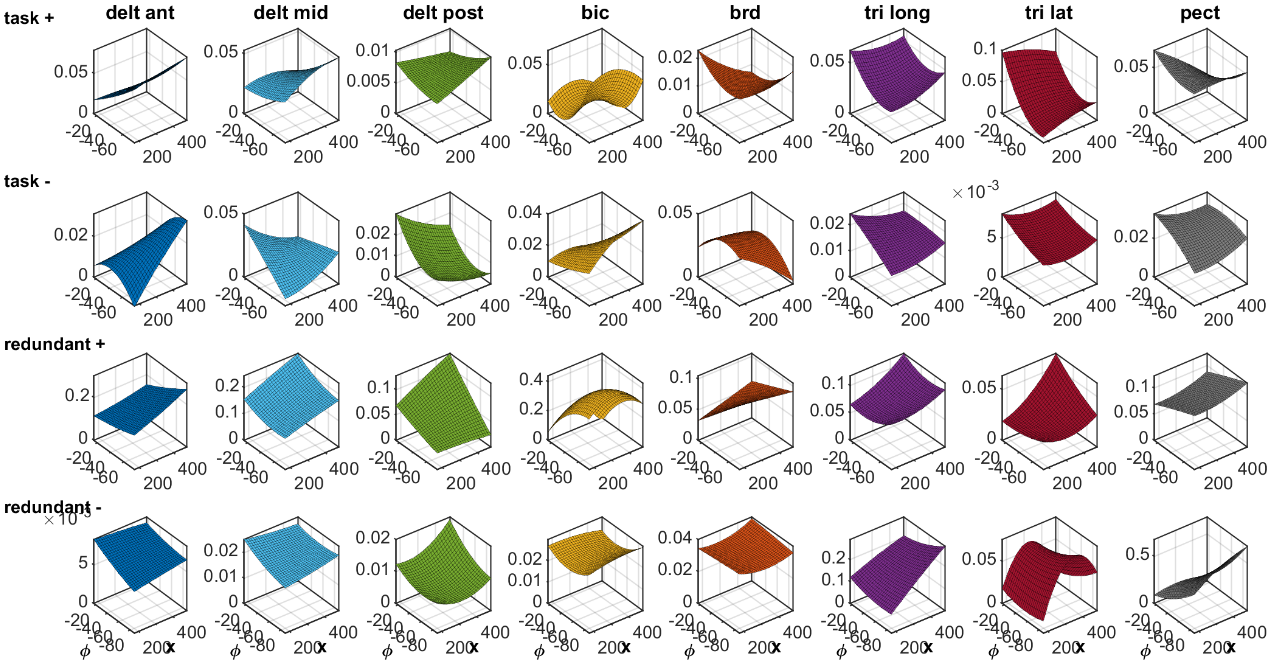

Figure 2: Subject #2's synergies.

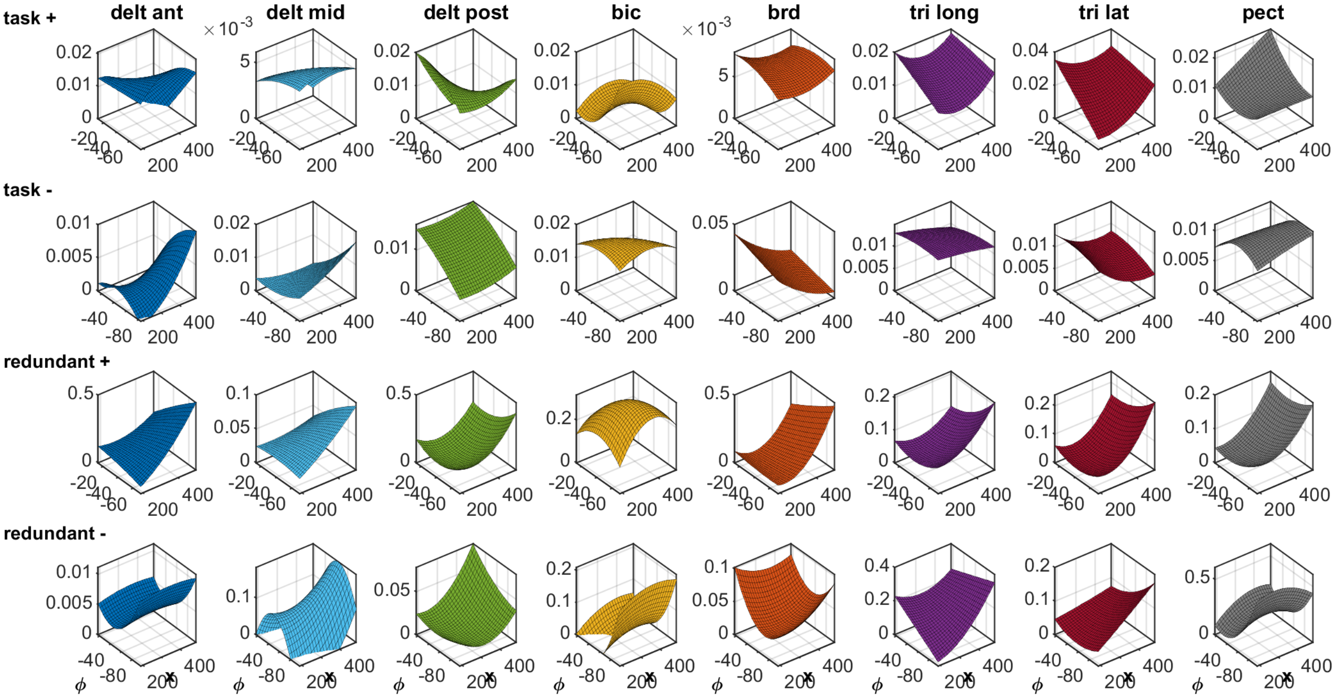

Figure 3: Subject #3's synergies.

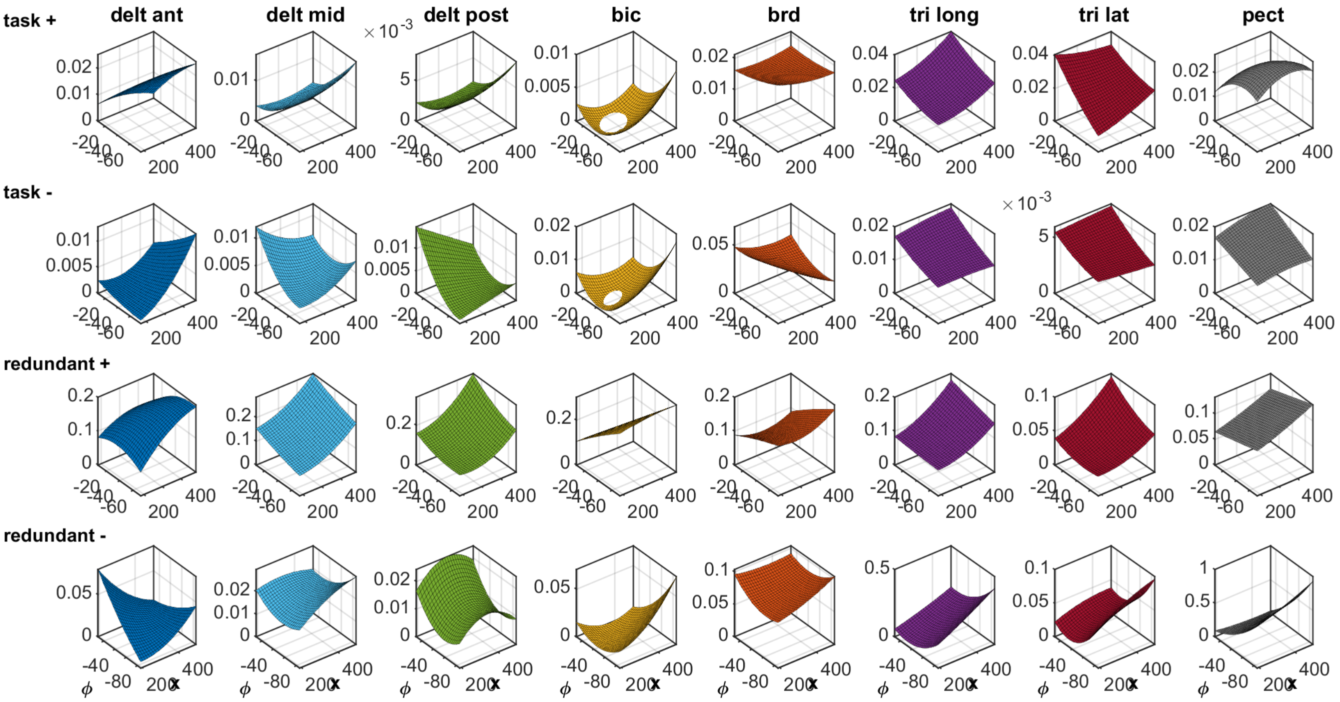

Figure 4: Subject #4's synergies.

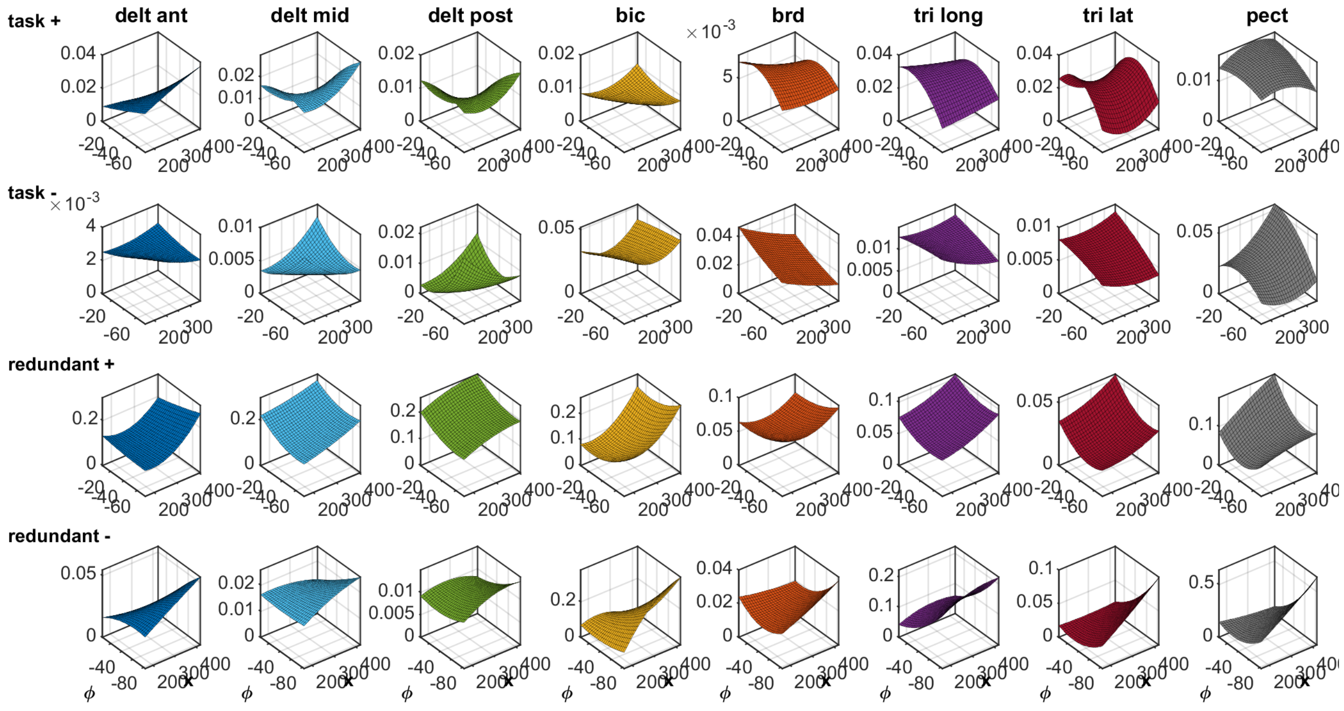

Figure 5: Subject #5's synergies.

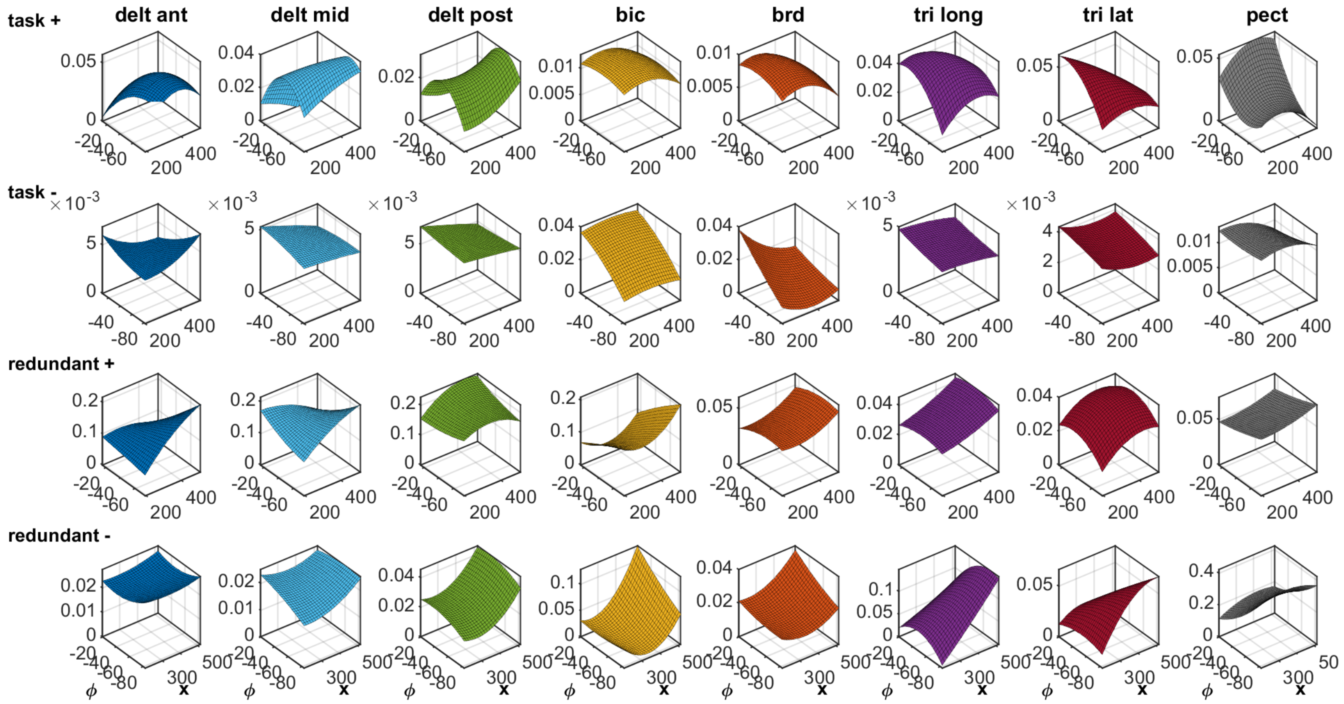

Figure 6: Subject #6's synergies.

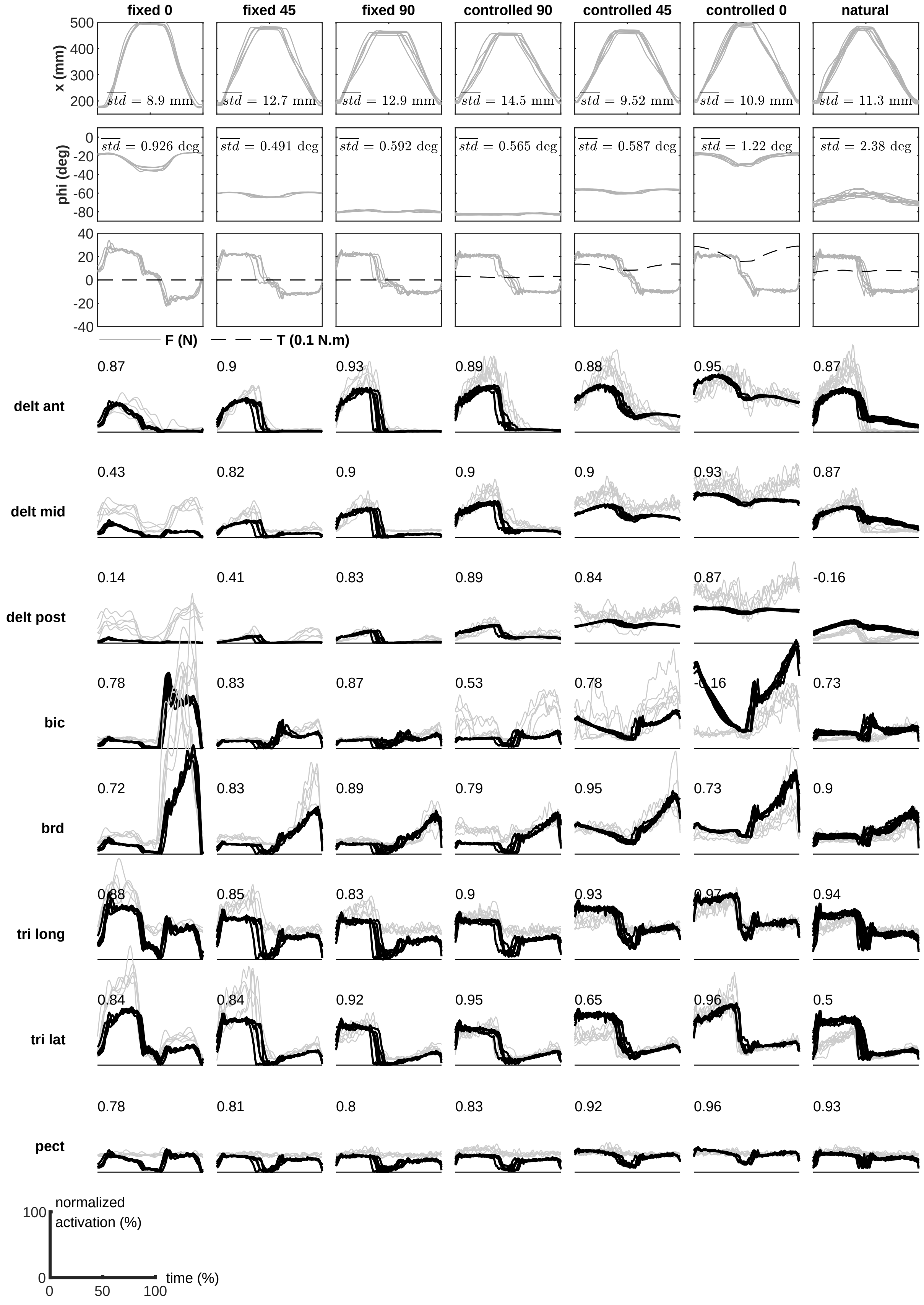

Figure 7: The motion trials for subject #1.

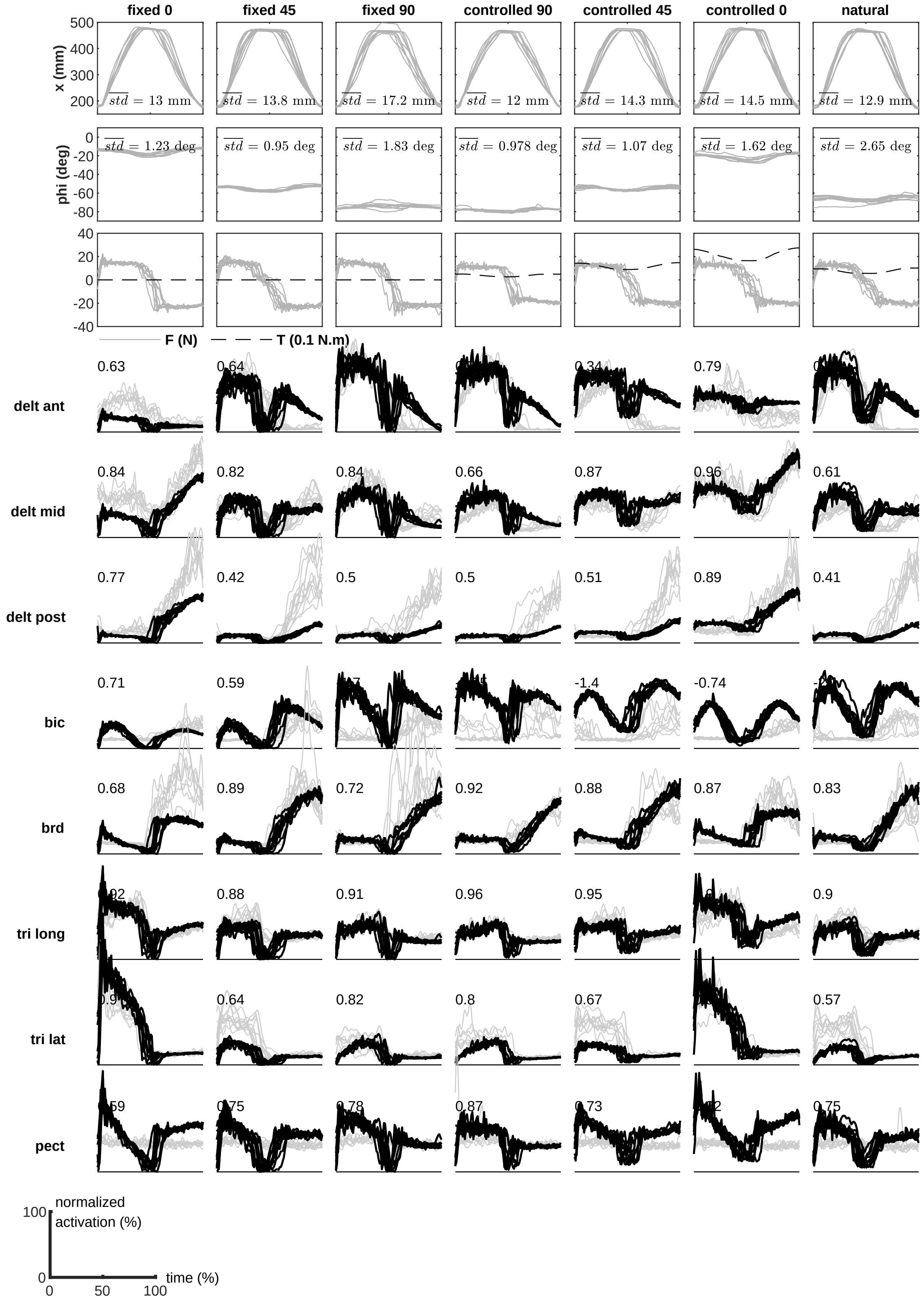

Figure 8: The motion trials for subject #2.

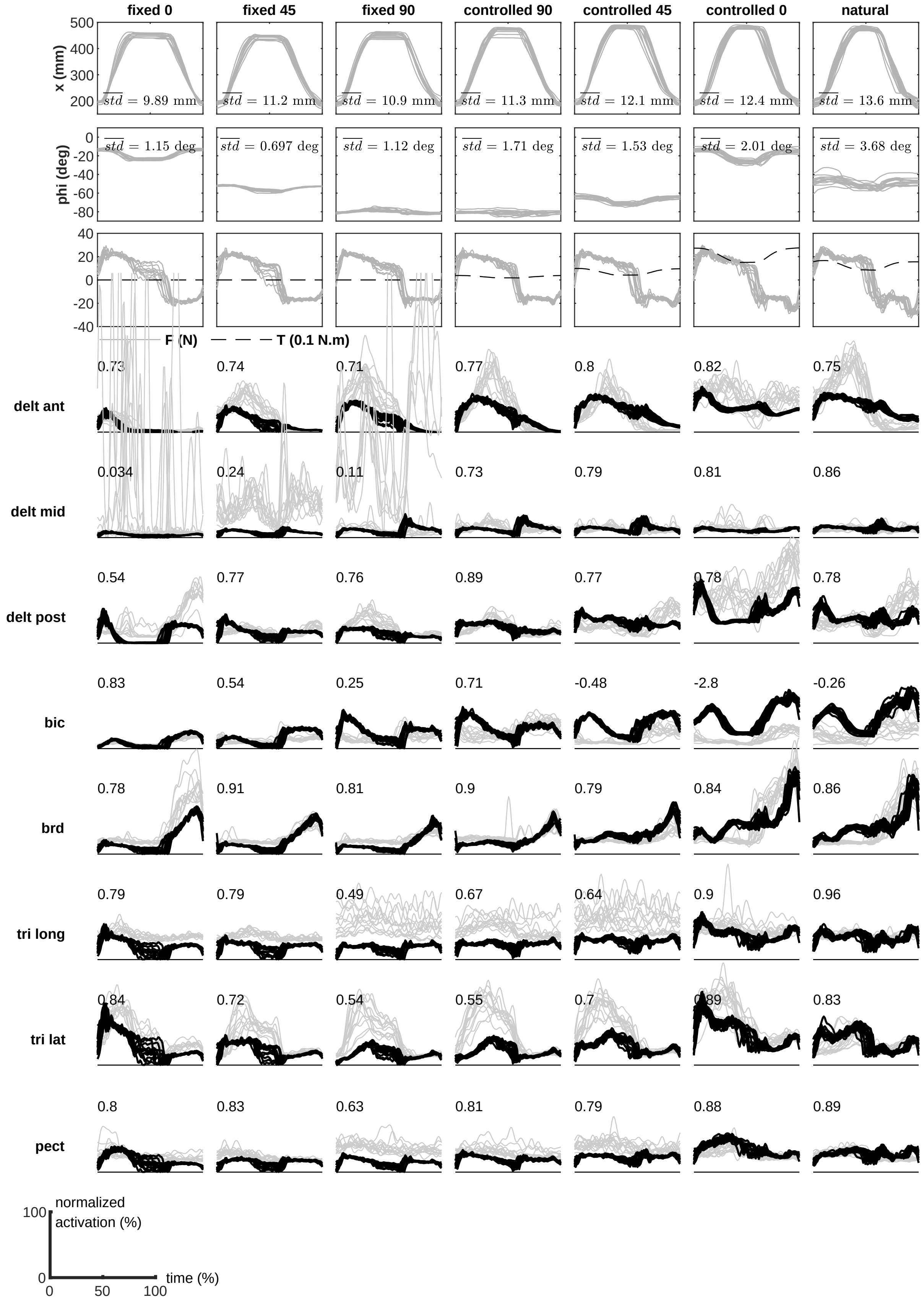

Figure 9: The motion trials for subject #3.

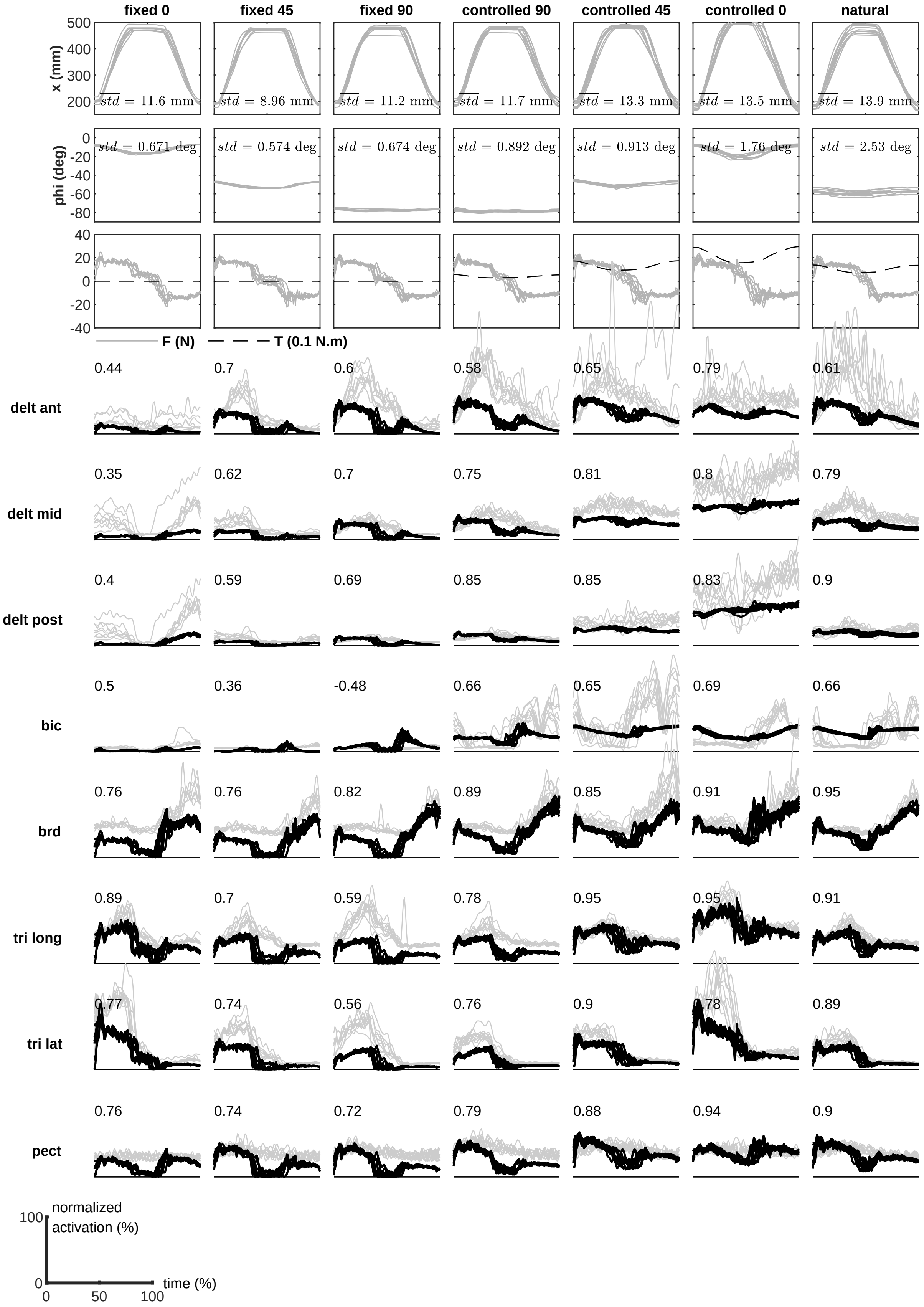

Figure 10: The motion trials for subject #4.

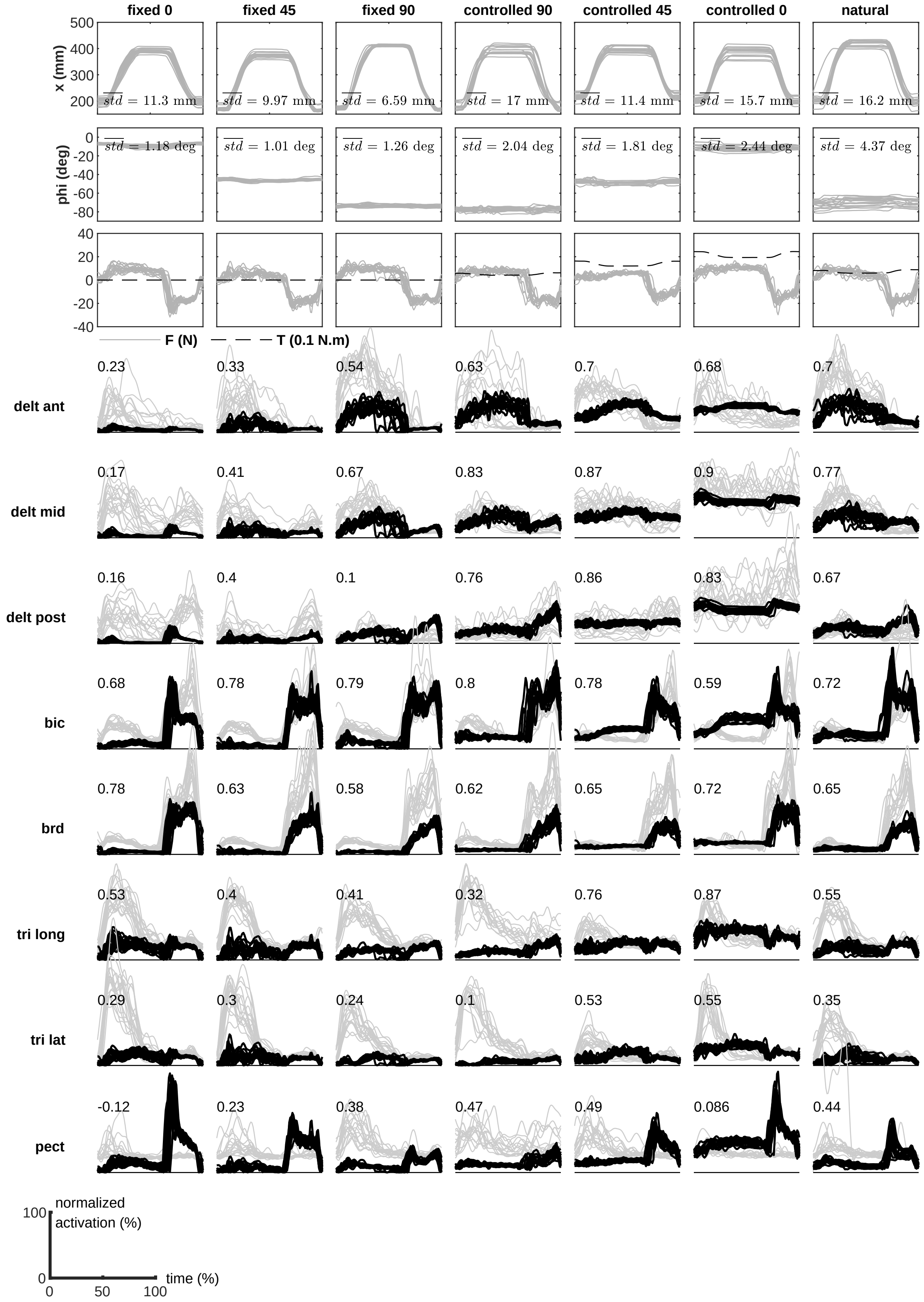

Figure 11: The motion trials for subject #5.

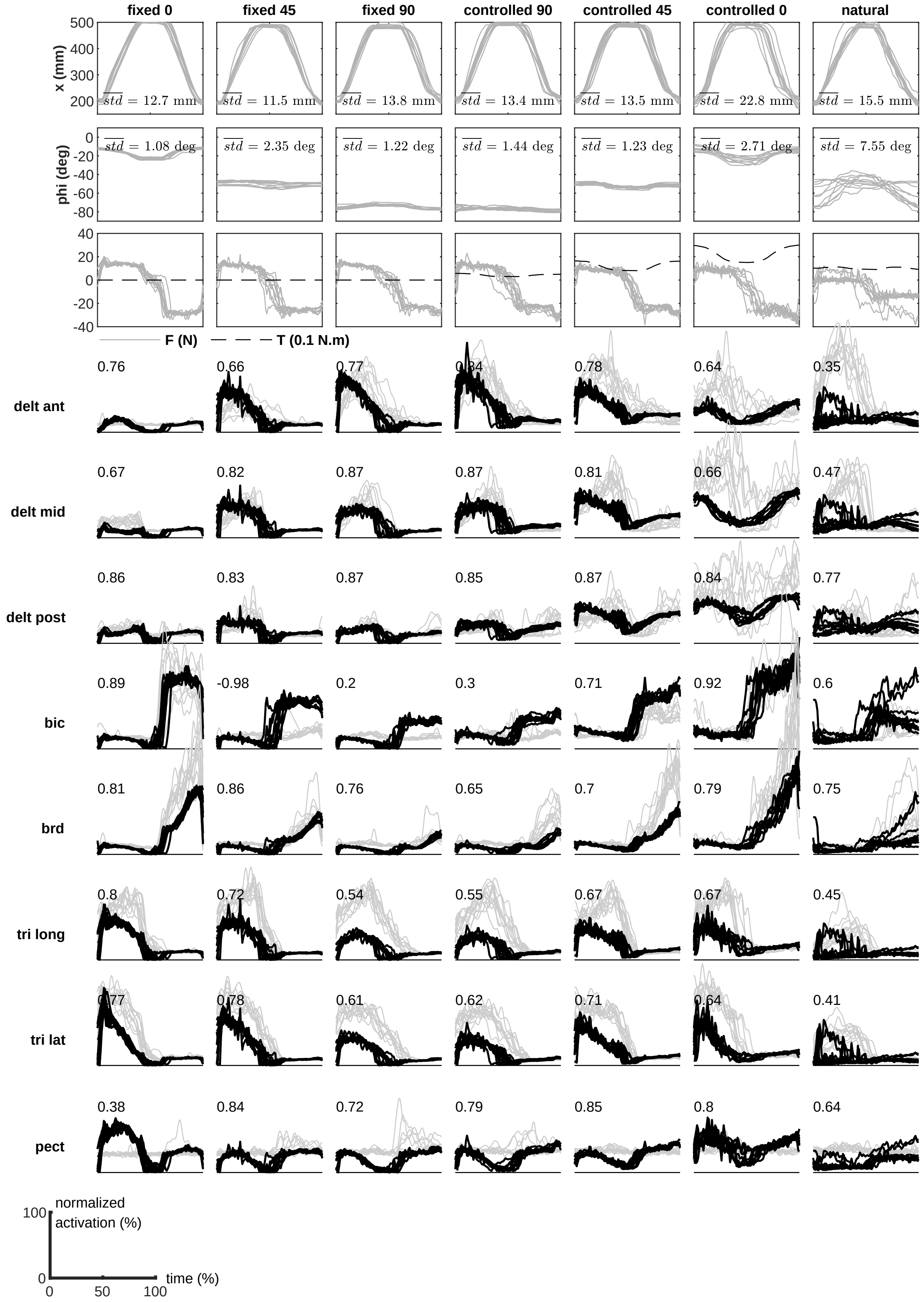

Figure 12: The motion trials for subject #6.
